# Genotoxic potential generated by biomass burning in the Brazilian Legal Amazon by Tradescantia micronucleus bioassay: a toxicity assessment study

**DOI:** 10.1186/1476-069X-10-41

**Published:** 2011-05-17

**Authors:** Herbert A Sisenando, Silvia R Batistuzzo de Medeiros, Paulo HN Saldiva, Paulo Artaxo, Sandra S Hacon

**Affiliations:** 1 Escola Nacional de Saúde Pública - ENSP, Fiocruz, Rio de Janeiro, CEP: 21041-210, RJ, Brazil; 2 Departamento de Biologia Celular e Genética, UFRN, Natal, CEP: 59072-970, RN, Brazil; 3 Departamento de Patologia, USP, São Paulo, CEP: 01246-903, SP, Brazil; 4 Departamento de Física Aplicada, USP, São Paulo, CEP: 05508-900, SP, Brazil; 5 Departamento de Patologia, UFF, Niterói, CEP: 24033-900, RJ, Brazil

## Abstract

**Background:**

The Brazilian Amazon has suffered impacts from non-sustainable economic development, especially owing to the expansion of agricultural commodities into forest areas. The Tangará da Serra region, located in the southern of the Legal Amazon, is characterized by non-mechanized sugar cane production. In addition, it lies on the dispersion path of the pollution plume generated by biomass burning. The aim of this study was to assess the genotoxic potential of the atmosphere in the Tangará da Serra region, using *Tradescantia pallida *as *in situ *bioindicator.

**Methods:**

The study was conducted during the dry and rainy seasons, where the plants were exposed to two types of exposure, active and passive.

**Results:**

The results showed that in all the sampling seasons, irrespective of exposure type, there was an increase in micronucleus frequency, compared to control and that it was statistically significant in the dry season. A strong and significant relationship was also observed between the increase in micronucleus incidence and the rise in fine particulate matter, and hospital morbidity from respiratory diseases in children.

**Conclusions:**

Based on the results, we demonstrated that pollutants generated by biomass burning in the Brazilian Amazon can induce genetic damage in test plants that was more prominent during dry season, and correlated with the level of particulates and elevated respiratory morbidity.

## Background

The Amazon is located in the northern portion of South America and 85% of its area lies within Brazilian territory, where it is known as "Brazilian Amazon Region" and accounting for 61% of the country's area. The region has been negatively affected by advancing economic development, especially agribusiness and cattle raising. This has provoked changes in soil pattern use, resulting in increased deforestation and an increase in biomass burning in both native forest and pasture areas [1,2]. Sugar cane is an example of agribusiness that is in full expansion in the Amazon biome, driven by the political incentive to produce and consume biofuel both nationally and internationally. Brazil is one of the largest producers of biofuel worldwide, with most production concentrated in the Midwest region. Sugar cane straw burning at harvest time is widely used in Brazilian production to facilitate harvesting and increase the yield of manual cutting; however, this archaic procedure results in increased pollutant concentration in the atmosphere [3-6].

The Amazon accounts for 62% of the burnings that occur in Brazil during the dry and intermediate, dry and rain periods. The southern region of the Amazon had the largest number of biomass burning sites in the period from 2004 to 2007 [7]. Biomass burning is an organic matter combustion process, characterized by the release of different toxic compounds into the atmosphere. These include carbon monoxide, nitrogen oxides, sulfur oxides, particulate matter and polycyclic aromatic hydrocarbons (PAHs), as well as the formation of ozone as a secondary pollutant [8-12].

In terms of the harm to human health associated with exposure to biomass burning pollutants, it is known that children, the elderly and individuals with previous cardiorespiratory diseases, including asthmatics, are the most susceptible to the effects of exposure to air pollution. Respiratory diseases are part of a group of more easily identified consequences; however, pollution may trigger cardiovascular diseases and other disorders, especially in children [13-17]. Studies have demonstrated that an increase in air pollution levels is associated to a rise in the number of hospitalizations for respiratory diseases [18-21].

The presence of a determinate pollutant or of a complex mixture may have the capacity, at high concentrations or after long exposure, of inducing genotoxic effects not only in humans, but also in animals, plants and bacteria, possibly compromising the health of ecosystems [22]. Among the tests used to assess the mutagenic potential of air pollutants are micronucleus bioassays with plants. This type of assay was first used by Evans [23] in *in vitro *experiments with the *Vicia faba *root, and is now widely used in studies aimed at environmental monitoring through the use of other plant models such as *Allium cepa *and *Tradescantia sp *[24]. The micronucleus test in *Tradescantia pallida *(Trad-MCN) is considered a valuable tool by many researchers, due to the simplicity of the methodology and sensitivity of this plant to genotoxic agents [25]. Micronuclei are structures resulting from whole chromosomes or chromosomal fragments that are lost during cell division and, for this reason, are not included in the nucleus of daughter cells, remaining in the cytoplasm of interphase cells, allowing us to detect the action of clastogenic and aneugenic agents [26]. The relationship between exposure to air pollution and micronucleus synthesis was described by Souza-Lima et al. [27] in a study with ozone, by Alves et al. [28] in a study with PAH and by Carvalho-Oliveira et al. [29] in work involving PM_2.5_.

The aim of this study was to assess the genotoxicity potential of biomass burning pollutants using *Tradescantia pallida *in two exposure models, associating micronucleus frequency with pollutant concentrations in the region and with the rate of hospitalization for respiratory diseases in children in 2008-2009.

## Methods

### Study region

**Figure 1:**
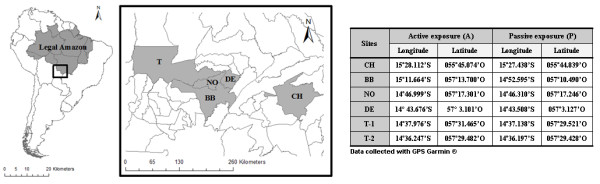
**Spatial distribution of the study site, delimiting the southern region of the Legal Amazon and the cities studied**. Georeferencing of sampling stations for the Tradescantia-micronucleus (Trad-MCN) assay.

The study was conducted in the Tangará da Serra microregion in a population of 152,422 inhabitants distributed over an area of 23,728,712 km^2^, located in the southern of the Legal Amazon, encompassing the municipalities of Barra do Bugres (BB), Denise (DE), Nova Olímpia (NO) and Tangará da Serra (T-1 and T-2) (Figure 1). All cities received 1 monitoring station for the Tradescantia-micronucleus test except Tangará da Serra (T) that received 2 stations for being the main community in the microregion and shows the worst indicators of morbidity from respiratory diseases in the region [30,31]. The region is located in a transition area between the Amazon biome and the Cerrado, with typical cycles of drought and rain that alter air pollution levels, and lies in the dispersion path of the pollution plume resulting from burnings in the Legal Amazon and pollution emanating from neighboring countries [5,32,33]. The region is the largest sugar cane producer and contains the two largest plants in the southern of the Brazilian Amazon [34]. The municipality of Chapada dos Guimarães, Brazil (CH) was selected as control area in this study because of its better air quality. There is no industrial production or sugar cane burning and automobile traffic is light, compared to the other municipalities involved. It was also chosen for the similarities in meteorological variables (rainfall, temperature and humidity) compared to other communities involved in this study (Table 1).

**Table 1:** Distribution of environmental and meteorological variables at all sampling stations.

Variables		CH	BB	NO	DE	T
**PM _2.5 _(μg/m^3^)**	D	9.9 (1.0)	20.9 (2.1)	20.8 (2.1)	19.3 (2.0)	20.9 (2.1)
	R	5.0 (1.0)	8.9 (1.8)	8.4 (1.7)	7.2 (1.4)	7.4 (1.5)
	Ratio D/R	2.0	2.4	2.4	2.7	2.8

**Rainfall (mm/d)**	D	1.1 (1.0)	1.0 (0.8)	1.1 (0.9)	0.9 (0.8)	0.9 (0.8)
	R	9.8 (1.0)	11.1 (1.1)	11.7 (1.2)	11.6 (1.2)	12 (1.2)
	Ratio R/D	8.9	11.1	10.6	12.9	13.3

**Temperature (°C)**	D	25.9 (1.0)	25.4 (1.0)	25.1 (1.0)	25.4 (1.0)	24.6 (0.9)
	R	25.4 (1.0)	25.2 (1.0)	24.8 (1.0)	25.0 (1.0)	24.4 (1.0)
	Ratio D/R	1.0	1.0	1.0	1.0	1.0

**Humidity (%)**	D	51.2 (1.0)	55.7 (1.1)	56.3 (1.1)	55.3 (1.1)	57.4 (1.1)
	R	80.5 (1.0)	82.5 (1.0)	83.4 (1.0)	83.0 (1.0)	84.2 (1.0)
	Ratio R/D	1.6	1.5	1.5	1.5	1.5

D = data obtained in the dry season. R = data obtained in the rainy season. D/R = ratio between the dry and rainy season. R/D = ratio between the rainy and dry season. The data in parentheses represent the ratio between the data of each test site and the control site (CH).

### *Tradescantia pallida *micronucleus assay

In this study 2 types of assessment were used: Active and Passive. In active assessment, *T. pallida *were cultivated in vases (50 cm × 17 cm × 17 cm) containing organic soil, watered daily, as described by Meireles et al. [35] and Sisenando et al. [36]. After a plant adaptation period (3 months), the vases were placed in 6 sampling sites (Figure 1). Passive assessment was carried out at 6 points, using plants of the same species and that grew in public gardens in the cities evaluated in this work (Figure 1). The inclusion criteria for the passive assay points were the proximity to the points selected for active monitoring and the existence of cultivated plants for more than 1 year. Inflorescence collection was performed at the same time in the two assessment types, and during two different periods: the dry season (May/08 to October/08) and the rainy season (November/08 to April/09), in order to encompass the two climatic periods of the region. The inflorescences were fixed in ethanol-acetic acid solution (3:1) for 24 hours and transferred to a solution of 70% ethanol for storage [37]. The flower buds containing tetrads in their initial stages were dissected, mounted on slides and stained in 2% acetic-carmine, according to the protocol established by Ma et al. [37]. Analysis consisted of counting 300 cells in the tetrad stage per slide, totaling 3000 cells per sampling site, where mean micronucleus frequency (%MCN) was determined according to criteria adopted by Fenech [38].

### Environmental and health data collection

Data on hospitalizations for respiratory diseases (Chapter X/ICD-10) in the study region and in the control area, during the dry and rainy season, were obtained from databanks for the period of May/08 to April/09, available by the Ministry of Health of Brazil in relation to Hospitalization Authorizations (HA) of the Department of Informatics (website: http://www.datasus.gov.br). The standardized rate was calculated (number of hospitalizations/1000 inhabitants) for children (age < 5 years), using population data from the Brazilian Institute of Geography and Statistics [31]. The children age group selected for this study is likely to be susceptible to the harmful effects of exposure to air pollution and less influenced by possible confounding factors (Ex.: alcohol and/or cigarette consumption) [39,40].

The daily means of air pollutant (fine particulate matter - PM_2.5_) and of meteorological variables (rainfall, humidity and temperature) in the region under the direct influence of burnings and in the control area, during the rainy and dry seasons, were obtained from CATT-BRAMS (Coupled Aerosol and Tracer Transport model of the Brazilian Regional Atmospheric Modeling System) accessed through SISAM (System of Environmental Information Integrated to Environmental Health - 2009). CATT-BRAMS is a 3D Eulerian model that allows a simultaneous prognosis of the concentration of determinate air pollutants, consistent with the atmospheric state simulated by the BRAMS model (based on the RAMS model). A more detailed description of this model was presented by Freitas et al. [11,41,42].

### Statistical analysis

To assess statistically significant differences among the different test points compared to the control point, during each period, the Kruskal-Wallis test was used followed by the Dunn multiple comparisons test. The statistically significant differences between the responses obtained in the dry and rainy seasons were analyzed using the Mann-Whitney test. This same test was also used to assess the significance between the responses obtained in active and passive exposure at each sampling point and in both seasons.

Spearman's correlation was used to analyze the %MCN obtained in active and passive exposure, as well as environmental and health data in the study region. The data were tabulated and analyzed using Microsoft Office^® ^2007 and SPSS^® ^16.0.

## Results

Figure 1 shows the southern region of the Legal Amazon and the municipalities involved in this study, in addition to the georeferencing of all the points sampled in the Tradescantia-micronucleus (Trad-MCN) assay under both exposure situations.

Table 1 shows the mean concentration of the environmental (PM_2.5_) and meteorological (rainfall, temperature and humidity) variables modeled by CATT-BRAMS in all the municipalities studied. In relation to meteorological variables, we observed that the rainy season showed accumulated rainfall 10 times higher than that observed in the dry season. We observed no differences (ratio = 1) in air temperature between the dry and rainy seasons, but relative air humidity in the rainy period was on average 1.5 times higher. When we compared the temperature and humidity data of each season with those of the control, we observed that the ratios varied between 0.9 and 1.1, suggesting no difference between the data. With respect to environmental variable, we found that PM_2.5 _demonstrated a profile in which the concentrations modeled in the dry season were about twice as high as the rainy season. When we compare the ratios obtained by dividing the data of each air pollutant exposure point by those obtained in the control, it can be observed that the exposure points were almost twice as large in the dry season and 1.5 times in the rainy season.

**Figure 2:**
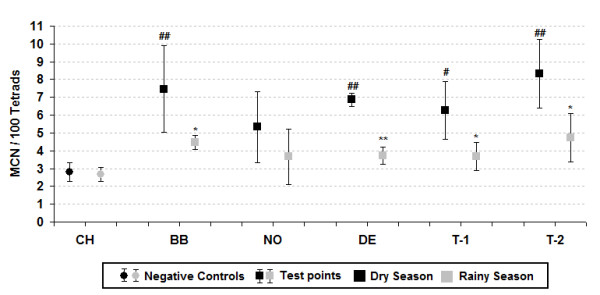
**Active exposure, micronuclei frequency in *T. pallida *at the study sites**. ^#^*p *< 0.05 and ^##^*p *< 0.01 by Kruskal-Wallis. *p < 0.05 and **p < 0.01 by Mann-Whitney.

**Figure 3:**
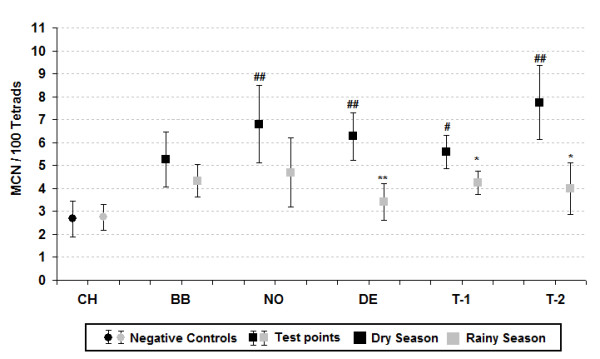
**Passive exposure, micronuclei frequency in *T. pallida *at the study sites**. ^#^*p *< 0.05 and ^##^*p *< 0.01 by Kruskal-Wallis. *p < 0.05 and **p < 0.01 by Mann-Whitney.

Mean micronucleus frequency (%MCN) in the active and passive exposures during the different periods is shown in Figures 2 and 3 respectively. The results of the two figures show that the %MCN obtained in the dry season were higher than those obtained in the rainy period. Statistically significant differences occurred in 58% of the sampling stations, especially the Denise and Tangará stations. A comparison between the micronucleus frequency of the test stations and the control station shows statistically significant differences in nearly all the comparisons during the dry season, unlike the rainy season, which exhibited no significant difference.

**Figure 4:**
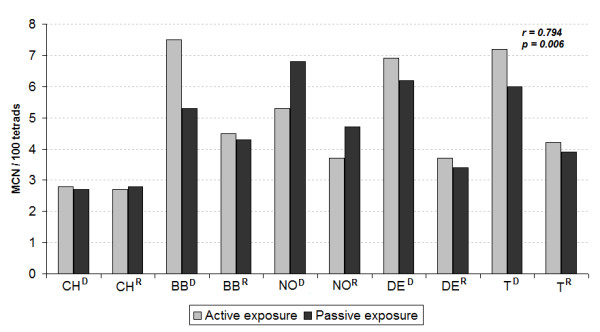
**Micronucleus frequencies in *T. pallida *in active and passive monitoring**. D = dry season. R = rainy season. Spearman's correlation coefficient (*r *and *p*) for the two types of exposure.

Figure 4 shows the distribution of %MCN in the municipalities and a comparison of the responses of the different types of exposure and periods sampled. No statistically significant difference was found between the two types of exposure, although we observed a greater response in terms of the increased number of micronuclei, in active exposure compared to passive, regardless of the period. When the two types of exposure are correlated using Spearman's method, a statistically significant positive correlation was observed (*r *= 0.794 *p *= 0.006).

Table 2 shows Spearman's correlation coefficient between the %MCN of each study area and their respective environmental and health data. Statistically significant positive correlations were detected between the %MCN and the means of the fine particulate matter - PM_2.5 _(*r *= 0.818 *p *= 0.004) and the adjusted rate of hospital morbidity from respiratory diseases in children - RD-C (*r *= 0.721 *p *= 0.019). A substantial negative relationship was observed between micronucleus frequency and mean accumulated rainfall (*r *= -0.636 *p *= 0.048). No significant associations were found between micronucleus frequency and the other meteorological variables (temperature and humidity). An extremely significant correlation was observed between the means of fine particulate matter in relation to accumulated rainfall (*r *= -0.806 *p *= 0.005).

**Table 2:** Correlation coefficients (and corresponding significance level) between the average micronucleus frequency of the sampling stations and the corresponding environmental and health data.

Spearman's Correlation	MCN ^a^	PM_2.5 _(μg/m^3^)	Rainfall (mm/d)	Temperature (°C)	Humidity (%)	RD-C ^b^
**MCN ^a^**	1.000	.818**	-.636*	-.030	-.297	.721*
	.	.004	.048	.934	.405	.019

**PM_2.5 _(μg/m^3^)**		1.000	-.806**	.176	-.612	.467
		.	.005	.627	.060	.174

**Rainfall (mm/d)**			1.000	-.418	.806**	-.152
			.	.229	.005	.676

**Temperature (°C)**				1.000	-.806**	-.467
				.	.005	.174

**Humidity (%)**					1.000	.236
					.	.511

**RD-C ^b^**						1.000
						.

a = average micronuclei frequency under active exposure. b = adjusted rate of hospital morbidity from respiratory diseases in children (Chapter X/ICD-10). * Correlation is significant at the 0.05 level (2-tailed). ** Correlation is significant at the 0.01 level (2-tailed). Data obtained in the dry and rainy period.

## Discussion

The risk attributed to exposure to low levels of air pollution for human pathogenesis using only epidemiological tools requires long-term studies and the participation of a large number of individuals exposed to pollutants. This type of study has disadvantages such as the high operational cost and logistical complexity and because if the association is established, a considerable number of participants will already have developed the disease. Although plant mutagenesis and aggravated human health cannot be compared, the genotoxicity assay with *T. pallida *can be used as a screening tool to assess human risk under unfavorable environmental conditions [43].

With respect to environmental variables, studies by Alves et al. [28], Isidori et al. [22], Klumpp et al. [44] and Savóia et al. [45] show that air temperature may act as a possible confounding factor, promoting the increase in micronucleus frequency. In our study, this confounding factor was minimized by virtue of the climatological homogeneity of the points. This can be observed by the fact that the ratio between the temperature of each test station and that obtained in the control station ranged between 0.9 and 1.0. The micronucleus frequency found in the actively and passively exposed plants in the control area of Chapada dos Guimarães was similar to that observed in the control group of studies that also assessed the genotoxicity potential of compounds present in air pollution, conducted by Batalha et al. [46] and Guimarães et al. [25] in Brazil, Carreras et al. [43] in Argentina, and Prajapati & Tripathi [47] in India.

The comparative results between the environmental and meteorological variables (Tab. 1) at the different points and exposure periods corroborate those reported by Rosa et al. [33] about the delimitation of two well-defined climatic periods (dry and rainy). This fact interferes significantly in the number of biomass burning points and pollutant dispersion, given that the pollution generated by biomass burning in the Amazon reaches its peak during the dry season [7]. Studies conducted by Andrade Júnior et al. [48], Savóia et al. [45] and Souza Lima et al. [27] to assess the genotoxicity of pollutants present in the atmosphere, using the Trad-MCN test also adopted a similar exposure sampling period. Although the literature contains studies that were conducted with much shorter exposure periods, the results remained significant [43,47,49]. In relation to micronucleus frequency, the results show that the response obtained in the dry season was higher than those in the rainy season at 92% of the points and statistically significant at most of them, irrespective of exposure type. These findings may be explained by the fact that in the dry season there are a larger number of hotspots generated by biomass and sugar cane straw burning and thus higher levels of air pollutants, especially fine particulate matter. Regardless of the type of exposure, when we compare the results obtained at the test points compared to control, we observe that, unlike the dry season, there are no significant differences in the rainy period. This may be owing to the lower number of burnings in this period [7], which generates a lower concentration of pollutants emitted into the troposphere and, associated to greater pollutant dispersion [50] during the rainy period, produced a reduction in %MCN at all the test points, in both exposures (active and passive).

When we analyze only %MCN of the dry season, we observe that stations DE, T-1 and T-2 showed the most important results, given that they were all statistically significant in both types of exposure. The response exhibited at station DE may be directly related to the proximity of the largest sugar cane plant of the region and to the fact that the municipality is the second largest sugar cane producer in the area, which contains large plantations surrounding the city limits where non-mechanized harvesting is still practiced [34]. The results of stations T (T-1 and T-2), both for micronuclei and pollutant levels, corroborate Ignotti et al. [30] and Rosa et al. [33], whose studies show that Tangará da Serra exhibits one of the worst indicators of morbidity and mortality from respiratory diseases in the southern region of Brazilian Amazon.

A comparison of %MCN obtained in the two types of exposure shows a very strong and statistically significant correlation (*r *= 0.794 *p *= 0.006), although there is an underestimation of passive compared to active response, in relation to the genotoxicity potential generated by air pollution. Other studies that used these two types of exposure simultaneously also obtained similar results when they evaluated the genotoxicity potential generated by pollutants emitted by the burning of fossil fuels in the cities of Feira de Santana/Brazil [35] and São Paulo/Brazil [25] using *T. pallida*. Meireles et al. [35] suggested that the difference between the two types of exposure may be due to an adaptation process of the plant, under passive exposure, to air pollutants at the exposure site. This likely adaptation of Tradescantia to air pollution was explained by Alves et al. [51], in a study conducted in São Paulo, Brazil, in which the reduction in stomata size on the abaxial surface of the leaves (region sensitive to air pollutants) after exposure to air pollutants, which provokes a reduction in gas exchange capacity and consequently in the possible effects that pollution has on the plant.

In terms of public health, the particulate matter is an important pollutant emitted into the atmosphere. Although the concentrations of fine particulate matter obtained in this study have been below the level suggested by the World Health Organization who is 25 μg/m^3^/24 h, it is important to report that WHO in its Air Quality Guidelines show that the continuous exposure PM_2.5 _concentrations even below the level suggested may increase the risks to the population exposed [52]. Studies conducted by Annesi-Maesano et al. [53] in France, Braga et al. [54] and Cançado et al. [20] in Brazil, Casas et al. [55] in Chile and Rojas-Martinez et al. [56] in México report a significant positive relationship between exposure to air pollutants and the increasing incidence of aggravated health in children. The main risk factor from this exposure is the capacity that these pollutants have to compromise pulmonary development [57], which causes impaired lung growth in children, increasing the risk of developing chronic obstructive pulmonary disease at adult age, as well as increased morbidity and mortality from cardiovascular diseases [56].

When we correlated the values of PM_2.5 _during the entire period with the %MCN of active exposure, we observed a positive and extremely significant correlation (*r *= 0.818 *p *= 0.004), corroborating the results obtained by Batalha et al. [46], Carvalho-Oliveira et al. [29], Carreras et al. [43] and Prajapati & Tripathi [47]. All these results show the importance of this biomarker in the evaluating genotoxic damage caused by exposure to particulate matter generated by burning biomass. The fact that we observed no significant correlation between %MCN and air temperature (*r *= -0.30 *p *= 0.934), contrasts with work published by Isidori et al. [22], Klumpp et al.[44] and Savóia et al. [45]. This may be owing to the similarity in temperature data from all the points, regardless of the period, a situation that reduces the confounding factor. When we correlated rainfall with PM_2.5 _data (*r *= -0.806 *p *= 0.005), we found a significant negative relationship, which shows that rain has a strong influence of biomass burning in the region. Indirectly, we observed that rainfall also has an important influence on micronucleus indices (*r *= -0.636 *p *= 0.048), since it reduces the number of burning points and pollutants emitted into the air.

The fact that we observed no statistically significant correlation between RD-C and the fine particulate matter (*r *= 0.467 *p *= 0.174) does not rule out the capacity of the latter to compromise the health of sensitive individuals. Mazzoli-Rocha et al. [58], in a study conducted with BALB/c mice exposed to particulate matter from sugar cane burning in Araraquara, Brazil, observed that the particulate matter collected in filters may induce alterations in the mechanics and pulmonary histology of the exposed mice, causing an increase in polymorphonuclear (PMN) cells and a reduction in mononuclear (MN) cells in the pulmonary parenchyma compared to the control.

A study carried out by Mariani et al. [49] in São José dos Campos, Brazil showed that micronucleus frequency in *T. pallida *exposed to air pollution was significantly associated to the mortality rate (deaths/10,000 inhabitants) adjusted for cardiovascular diseases (*r *= 0.841 *p *= 0.036) and cancer (*r *= 0.890 *p *= 0.018) in the general population. The authors showed that there was a substantial relationship, albeit not significant, between micronucleus indices and mortality from chronic obstructive pulmonary disease (*r *= 0.640 *p *= 0.170). These data corroborate the result obtained for the relationship between the standardized rate (hospital morbidity/1000 inhabitants) in children (RD-C) and %MCN (*r *= 0.721 *p *= 0.019), showing a strong, statistically significant positive correlation between the two variables.

A factor that could be considered a limitation of this study was the number of vases with *T. pallida *at each point of the active exposure, which limited the number of inflorescences analyzed by point and that may have contributed to the large standard deviation in some stations. Another limitation was the analysis of all Chapter X/ICD-10 for construction of the adjusted morbidity rate in children since not all diseases in this chapter are related to exposure to air pollutants. However, this measure was necessary to minimize failures in the procedures for notification of diseases by the health system observed by Rosa et al. [33] in a study that analyzed the magnitude of the hospital admissions for respiratory diseases in the region. This study observed that the increase in notifications due to pneumonia can be explained by the higher financial remuneration of this type of procedure as compared with hospitalizations for asthma. Another important factor noted by Rosa et al. [33] and that influencing the clinical diagnosis of asthma in the region was the prejudice of the parents in accepting that the child has the disease.

## Conclusions

The present work is a pioneer study on the assessment of genotoxic potential caused by biomass burning in the Legal Amazon region, Furthermore, it showed that in situ biomonitoring using a sensitive organism (*Tradescantia pallida *Purpurea) may be an important tool for monitoring air quality in regions that do not contain environmental monitoring stations. Considering that pollution generated by biomass burning is a complex mixture, it is difficult to attribute the increase in %MCN observed in our study to any toxic element integrated in the particular matters. However, the contribution of the present study lies the fact that we found significant relationship between the increase in micronucleus frequency and the increased exposure to fine particulate matter. It was also observed that this rise in micronucleus frequency was significantly correlated with the increase in the standardized rate of respiratory diseases in children, suggesting that research using *Tradescantia pallida *is an important biological indicator to be included in the assessment of human risk to exposure to toxic agents emitted by biomass burning.

## List of Abbreviations

DNA: Deoxyribonucleic Acid; PAHs: Polycyclic Aromatic Hydrocarbons; Trad-MCN: Micronucleus Test in *Tradescantia pallida; *PM_2.5_: Fine Particulate Matter; BB: Barra do Bugres, DE: Denise; NO: Nova Olímpia; T: Tangará da Serra; T1: Tangará da Serra - station 1; T2: Tangará da Serra - station 2; CH: Chapada dos Guimarães; %MCN: Mean Micronucleus Frequency; ICD-10: International Statistical Classification of Diseases - 10th Revision; HA: Hospitalization Authorizations; CATT-BRAMS: Coupled Aerosol and Tracer Transport Model of the Brazilian Regional Atmospheric Modeling System; SISAM: System of Environmental Information Integrated to Environmental Health; RD-C: Adjusted Rate of Hospital Morbidity from Respiratory Diseases in Children; PM: Particulate Matter; PMN: Polymorphonuclear; MN: Mononuclear Cells.

## Competing interests

The authors declare that they have no competing interests.

## Authors' contributions

HAS participated in the study design, conducted the analyses, and drafted the manuscript; SRBM participated in the study design, guided the genotoxicity analyses, and reviewed the manuscript drafts; PHNS guided the *Tradescantia pallida *assay, and reviewed the manuscript drafts; PA was involved in revision and preparation of the manuscript for publication; SSH conceived of the study, participated in its design and coordination and helped to draft the manuscript. All authors read and approved the final manuscript.
